# Lateral neck dissection for the treatment of synchronous and metachronous lateral neck metastasis of N1b papillary thyroid cancer

**DOI:** 10.3389/fendo.2023.1166640

**Published:** 2023-06-22

**Authors:** Hyeok Jun Yun, Jin Seok Lee, Jun Sung Lee, Seok Mo Kim, Hojin Chang, Yong Sang Lee, Hang-Seok Chang, Cheong Soo Park

**Affiliations:** ^1^ Department of Surgery, Institute of Refractory Thyroid Cancer, Thyroid Cancer Center, Gangnam Severance Hospital, Yonsei University College of Medicine, Seoul, Republic of Korea; ^2^ Department of Surgery, CHA Ilsan Medical Center, Goyang-si, Republic of Korea

**Keywords:** metachronous lateral neck dissection metachronous lateral neck dissection, synchronous lateral neck dissection, lateral neck dissection, papillary thyroid cancer, head and neck neoplasms

## Abstract

**Introduction:**

Metachronous lateral neck recurrence after thyroidectomy for N1b papillary thyroid cancer is accompanied by high morbidity and increased difficulty of reoperation. From the perspective of recurrence, the objective of this study was to compare patients who underwent metachronous lateral neck dissection (mLND) despite initial thyroidectomy and patients who underwent synchronous lateral neck dissection (sLND) for papillary thyroid cancer and analyze the risk factors for recurrence after mLND.

**Method:**

This retrospective study involved 1,760 patients who underwent lateral neck dissection for papillary thyroid cancer at the Gangnam Severance Hospital, a tertiary medical center in Korea, from June 2005 to December 2016. The primary outcome was structural recurrence, and secondary outcome measures were risk factors of recurrence in the mLND group.

**Result:**

A total of 1,613 patients underwent thyroidectomy and sLND at diagnosis. In 147 patients, thyroidectomy alone was performed at the time of diagnosis, and mLND was performed when recurrence to the lateral neck lymph node was confirmed. During a median follow-up of 102.1 months, 110 (6.3%) patients experienced a recurrence. There was no significant difference in the recurrence between the sLND and mLND groups (6.1% vs 8.2%, P=.32). The period from lateral neck dissection to recurrence was longer in the mLND group than in the sLND group (113.6 ± 39.4 months vs 87.0 ± 33.8 months, respectively, P<.001). Age ≥50 years (adjusted HR=5.209, 95% CI=1.359–19.964; P=.02), tumor size >1.45 cm (adjusted HR=4.022, 95% CI=1.036–15.611; P=.04), and lymph node ratio in the lateral compartment (adjusted HR=4.043, 95% CI=1.079–15.148; P=.04) were independent variables predictive of recurrence after mLND.

**Conclusion:**

mLND is suitable for treating lateral neck recurrence in patients with N1b papillary thyroid cancer who previously underwent thyroidectomy. Lateral neck recurrence after treatment in patients who underwent mLND was predicted by age, tumor size, and lymph node ratio in the lateral compartment.

## 1 Introduction

A significant increase in the incidence of papillary thyroid cancer (PTC), mainly owing to the expansion of diagnostic imaging, has been reported worldwide (1–3). Although mortality associated with PTC remains low, it has been suggested that the ability to detect small, relatively indolent tumors by radiographic examination may be improved, which may lead to overdiagnosis (1, 4). However, data from the past decade have shown conflicting results. The incidence of advanced PTC is increasing, with the incidence-based thyroid cancer mortality increasing as well (2, 5). In addition, the diagnosis rate of low-risk thyroid cancer is increasing, which cannot be explained by the overdiagnosis alone (6).

The recent American Thyroid Association guidelines recommend lobectomy as the standard surgical treatment for low-risk PTC unless there are clear indications for removing the contralateral lobe (7). Moreover, most guidelines recommend that lateral neck dissection (LND) should only be performed with therapeutic intent when lymph node (LN) involvement is proven and should not be performed for prophylactic purposes (7, 8). Despite this consensus regarding its indications, the extension of therapeutic LND is still debated (9, 10). Thus, surgeons can determine the extent of surgery through careful preoperative radiological examination but cannot accurately assess the possibility of recurrence after surgery. Thyroid cancer recurrence is a highly unfavorable event for patients and surgeons owing to the difficulty of reoperation, high morbidity, and low quality of life (11). Therefore, patients with PTC who have undergone additional surgery for locoregional recurrence should be closely monitored, despite the strict criteria for thyroidectomy.

Here, recurrence rates among patients who underwent metachronous LND (mLND) during follow-up, despite initial thyroidectomy performed according to appropriate preoperative evaluation, were compared to those who underwent synchronous LND (sLND), and factors affecting recurrence in the mLND group were analyzed.

## 2 Materials and methods

### 2.1 Patients

**Figure 1:**
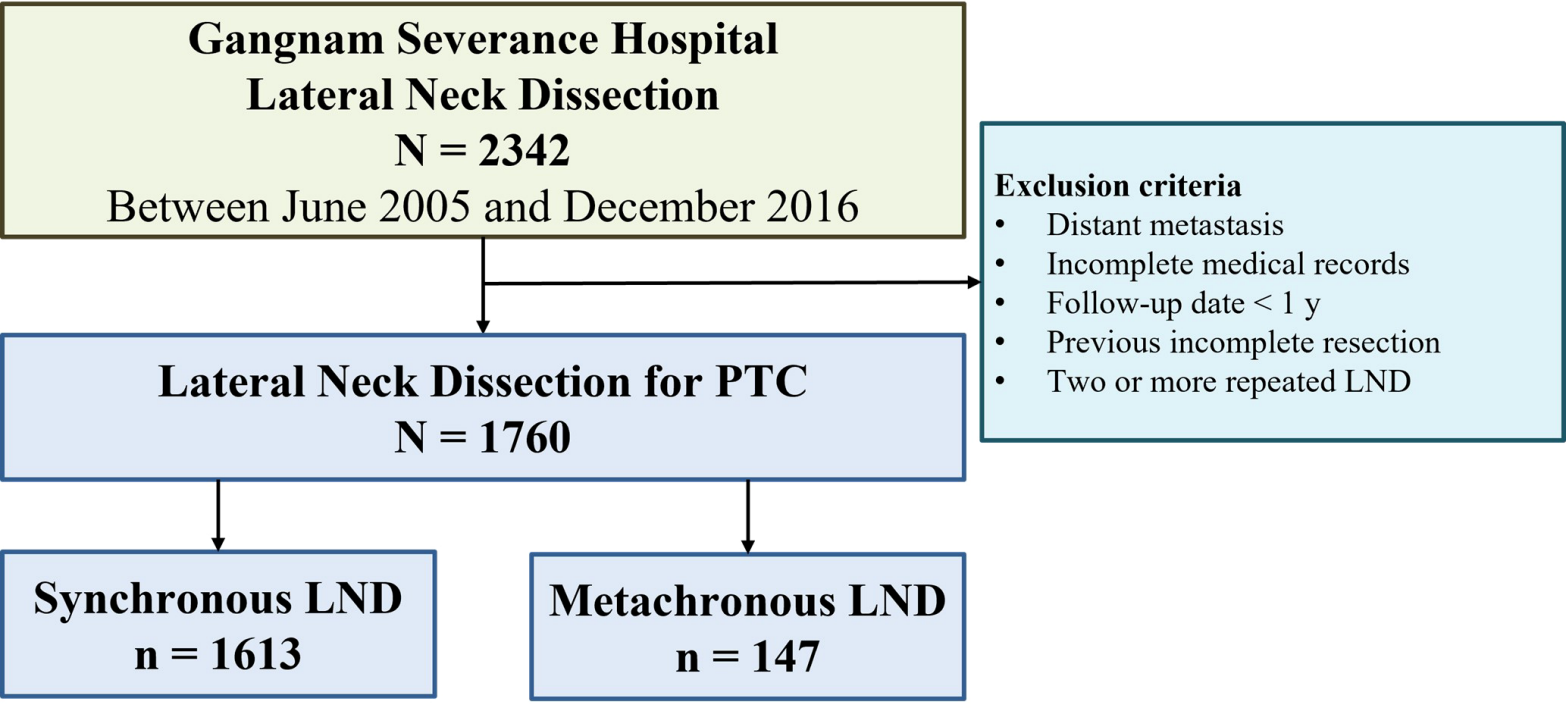
Flow diagram for patients with papillary thyroid cancer (PTC) who underwent lateral neck dissection (LND).

From June 2005 to December 2016, lateral compartment neck resection was performed on 2,342 patients at the Gangnam Severance Hospital Thyroid Cancer Center, Yonsei University College of Medicine, and the medical records of 1,760 of these patients were retrospectively analyzed (**Figure 1**). The exclusion criteria were as follows: 1) distant metastasis at diagnosis, 2) follow-up <1 year, 3) incomplete resections in previous surgeries, 4) two or more repeated LNDs, and 5) incomplete medical records.

The Institutional Review Board of Gangnam Severance Hospital, Yonsei University College of Medicine (Seoul, Korea) approved this study (approval number: 3-2021-0120), and the protocol complied with the tenets of the Declaration of Helsinki. Owing to the retrospective nature of the study, neither patient approval nor informed consent were required.

### 2.2 Preoperative evaluation

Preoperative thyroid ultrasonography (US), neck computed tomography (CT) scan, and thyroid function test were done routinely. Pathological confirmation of lateral neck metastasis in patients with mLND and sLND was performed by ultrasound-guided fine-needle biopsy when suspicious were found on US or CT scans. If suspected for distant metastasis, chest CT and positive emission tomography (PET)-CT were done. Basal complete blood count, prothrombin times, activated partial thromboplastin time, chemistry labs such as levels of blood urea nitrogen/creatinine, electrolyte, liver enzymes, and chest radiograph were obtained.

### 2.3 Surgery and follow-up

All included patients underwent lateral compartment neck dissection synchronously or metachronously with total thyroidectomy, including central compartment neck dissection. The lateral compartment neck dissection included cervical levels IIa–Vb. The spinal accessory nerve, internal jugular vein, and sternocleidomastoid muscle were generally preserved. Lateral compartment neck dissection encompasses the area bounded by the hypoglossal nerve superiorly, the subclavian vein inferiorly, and the trapezius muscle laterally. In addition, therapeutic central compartment neck dissection was performed in the area extending cranially to the superior thyroid arteries and pyramidal lobe, caudally to the innominate vein, laterally to the carotid sheath, and dorsally to the prevertebral fascia.

The patients who underwent sLND were simultaneously diagnosed with thyroid cancer and lateral neck metastasis. The sLND group underwent bilateral total thyroidectomy with central compartment LN dissection and unilateral/bilateral lateral compartment neck dissection. The mLND group underwent less than total or total thyroidectomy with or without central compartment LN dissection and then underwent unilateral or bilateral LND after lateral neck recurrence was detected during the follow-up period. The thyroid and neck dissection specimens were carefully examined in the operating room and then sent for pathological examination. Gross and microscopic findings of primary tumors and LNs were carefully noted.

All patients underwent postoperative radioactive iodine-131 (RAI) ablation therapy using 150–200 mCi, 1–2 months after the operation. The serum stimulated thyroglobulin and suppressed thyroglobulin levels were measured in all patients with an elevation of the thyroid-stimulating hormone (TSH, level > 70 IU/mL) before and after RAI ablation. Whole-body iodine scintigraphy was performed 2–7 days after initiating RAI ablation therapy. All patients were regularly followed up by neck ultrasonography, chest radiography, whole-body iodine scanning, and measurements of serum-free thyroxine, TSH, Tg, and anti-Tg antibody concentrations. Patients visited the outpatient clinics for regular clinical examinations every 3 months for the first year, every 6 months during the second year, and annually thereafter.

### 2.4 Recurrence

Recurrence was defined as the presence of tumors at locoregional and/or distant sites. Patients with suspected locoregional recurrence underwent fine-needle aspiration biopsy and imaging. Distant metastasis was diagnosed using whole-body iodine scintigraphy, chest CT, and PET-CT and confirmed by serial imaging and biopsy. Recurrence was defined as structural recurrence identified by imaging, regardless of serum Tg levels, but not as biochemical recurrence.

### 2.5 Variables

Demographic, operative, and pathological data were carefully reviewed. Operative findings and tumor specimens were investigated to determine tumor size, multifocality, extrathyroidal extension (ETE), and thyroiditis. In the mLND group, age, tumor characteristics, node, and metastasis (TNM) stages were confirmed at the time of first operation before LND. ETE included both microscopic ETE and gross ETE. Multifocality was defined as the presence of two or more isolated/non-contiguous tumor foci of any size in the resected thyroid gland. The numbers of LNs and the LN ratios were separately calculated in the central, lateral, and both compartments of the neck. In the mLND group that underwent completion thyroidectomy, the number of central LNs was the total number of operations.

### 2.6 Statistical analysis

The primary endpoint was structural recurrence of any lesion. We defined the recurrence-free survival (RFS) as the time from the first operation to the confirmation of recurrence. Continuous variables are represented as mean (standard deviation, SD) or median (range), while categorical variables are shown as number (percentage). Independent t-test and chi-square analyses were used to compare the sLND and mLND groups. The secondary endpoint was recurrence in the mLND group. Univariate and multivariate Cox-proportional hazards models were used to analyze the relationship between clinicopathological variables and RFS. Using backward elimination, variables with P<.10 in univariate analyses were selected for the multivariate Cox proportional hazards regression analyses. The estimated hazard ratios (HR) and 95% confidence intervals (CI) were calculated. We used the log-rank test and Kaplan–Meier curve to calculate differences in the RFS. The area under the receiver operating characteristics (ROC) curve (AUC) was also determined to estimate the RFS according to age at first thyroid surgery, tumor size, LN ratio in the central, lateral, and both compartments of the neck, and on/stimulated Tg levels. All tests were two-sided, and differences were statistically significant at P<0.05. We performed all statistical analyses using SPSS version 25.0 (IBM Inc., Armonk, NY, USA).

## 3 Results

### 3.1 Patients’ characteristics and pathology

This study included 1,760 patients (1,206 women, 554 men) with a mean age of 41.8 ± 12.4 years. Patient characteristics are shown in **Table 1**. The mean tumor size in patients with histologically proven PTC was 1.6 ± 1.1 cm; tumors >4 cm were found in 70 (4.0%) patients. Tumor multifocality was identified in 856 (48.6%) patients, and ETE in 1,392 (79.1%) patients. Histologically, chronic lymphocytic thyroiditis (CLT) was present in 614 (34.9%). The total number of retrieved central LNs was 9.5 ± 6.7, that of metastatic central LNs was 4.8 ± 4.7, that of retrieved lateral LNs was 40.8 ± 17.1, and that of metastatic lateral LNs was 6.2 ± 5.3.

**Table 1:** Clinicopathological characteristics of patients.

Variable	N = 1760 (%)
Age, year	41.8 ± 12.4
>45	592 (33.6)
Sex (Female)	1206 (68.5)
Tumor size, cm	1.6 ± 1.1
>4 cm	70 (4.0)
Tumor multifocality	856 (48.6)
Extrathyroidal extension	1392 (79.1)
Thyroiditis	614 (34.9)
T classification T1/T2/T3/T4a/T4b	309/45/1243/146/17 (17.6/2.6/70.6/8.3/1.0)
LND (ipsilateral/bilateral)	1529/231 (86.9/13.1)
Central LNs	
Retrieved	9.5 ± 6.7
Metastatic	4.8 ± 4.7
Lateral LNs	
Retrieved	40.8 ± 17.1
Metastatic	6.2 ± 5.3
Structural recur	110 (6.3)
Median follow-up (range), month	93.8 (12.3–233.1)

pTNM proposed by the American Joint Committee on Cancer (AJCC, 8th edition); LND, lateral neck dissection; LNs, lymph nodes.

### 3.2 Comparison of sLND and mLND

A comparison of sLND and mLND is presented in **Table 2**. The sLND group had 1,613 patients, and the mLND group had 147 patients. The mean age was significantly higher in the mLND group than in the sLND group (44.9 ± 11.8 years and 41.5 ± 12.4 years, respectively, P=.001). The proportion of women was significantly higher in the mLND group than in the sLND group (67.8% and 76.9%, respectively, P=.02). The tumor size was significantly larger in the sLND group than in the mLND group (1.6 ± 1.0 cm and 1.4 ± 1.2 cm, respectively, P=.001). Tumor multifocality was found in 49% and 44.9% of patients in the sLND and mLND groups, respectively. ETE was significantly greater in the sLND group than in the mLND group (80.8% and 59.9%, respectively, P<.001). In the pathological examination, CLT was more frequent in the sLND group than in the mLND group (36.0% and 22.4%, respectively, P=.001). Bilateral LND rates between the sLND and mLND groups did not differ significantly (13.1% and 13.6%, respectively, P=.86). The number of central LNs did not differ significantly between the groups. The number of retrieved lateral LNs was significantly greater in the sLND group than in the mLND group (41.1 ± 17.2 and 37.9 ± 15.3, respectively, P=.04), and the number of metastatic lateral LNs was significantly different (6.2 ± 5.5 and 5.2 ± 3.9, respectively, P=.002). The number of total retrieved LNs was significantly greater in the sLND group than in the mLND group (50.6 ± 19.8 and 45.4 ± 17.3, respectively, P=.002), and the number of total metastatic LNs was significantly greater in the sLND group than in the mLND group (11.1 ± 8.6 and 9.2 ± 7.1, respectively, P=.003).

**Table 2:** Comparison of the synchronous lateral neck dissection (sLND) and metachronous lateral neck dissection (mLND) groups.

Variable	sLND n = 1613	mLND n = 147	*P*-value
Age, mean ± SD, year	41.5 ± 12.4	44.9 ± 11.8	.001
Sex (female)	1093 (67.8)	113 (76.9)	.02
Tumor size, mean ± SD, cm	1.6 ± 1.0	1.4 ± 1.2	.02
Tumor multifocality	790 (49.0)	66 (44.9)	.34
Extrathyroidal extension	1304 (80.8)	88 (59.9)	<.001
Thyroiditis	581 (36.0)	33 (22.4)	.001
LND (unilateral/bilateral)	1402/211 (86.9/13.1)	127/20 (86.4/13.6)	.86
Central LNs			
Retrieved, mean ± SD, n	9.5 ± 6.6	8.6 ± 7.7	.11
Metastatic, mean ± SD, n	4.8 ± 4.7	4.7 ± 4.6	.76
Positive ratio, mean ± SD, n	0.50 ± 0.34	0.50 ± 0.38	.93
Lateral LNs			
Retrieved, mean ± SD, n	41.1 ± 17.2	37.9 ± 15.3	.04
Metastatic, mean ± SD, n	6.2 ± 5.5	5.2 ± 3.9	.002
Positive ratio, mean ± SD, n	0.15 ± 0.10	0.14 ± 0.09	.281
Total LNs			
Retrieved, mean ± SD, n	50.6 ± 19.8	45.4 ± 17.3	.002
Metastatic, mean ± SD, n	11.1 ± 8.6	9.2 ± 7.1	.003
Positive ratio, mean ± SD, n	0.21 ± 0.13	0.20 ± 0.12	.23
T classification			
T1/T2/T3/T4	261/40/1160/152 (16.2/2.5/71.9/9.4)	49/5/90/3 (33.3/3.4/61.2/2.0)	<.001
Structural recurrence	98 (6.1)	12 (8.2)	.32
period until recurrence, median (range), months	86.4 (5.9–200.8)	115.4 (18.4–233.1)	<.001
Median follow-up, median (range), months	97.8 (12.3–212.9)	130.5 (12.6–245.2)	<.001

pTNM proposed by the American Joint Committee on Cancer (AJCC, 8th edition); LND, lateral neck dissection; LNs, lymph nodes; SD, standard deviation.

**Figure 2:**
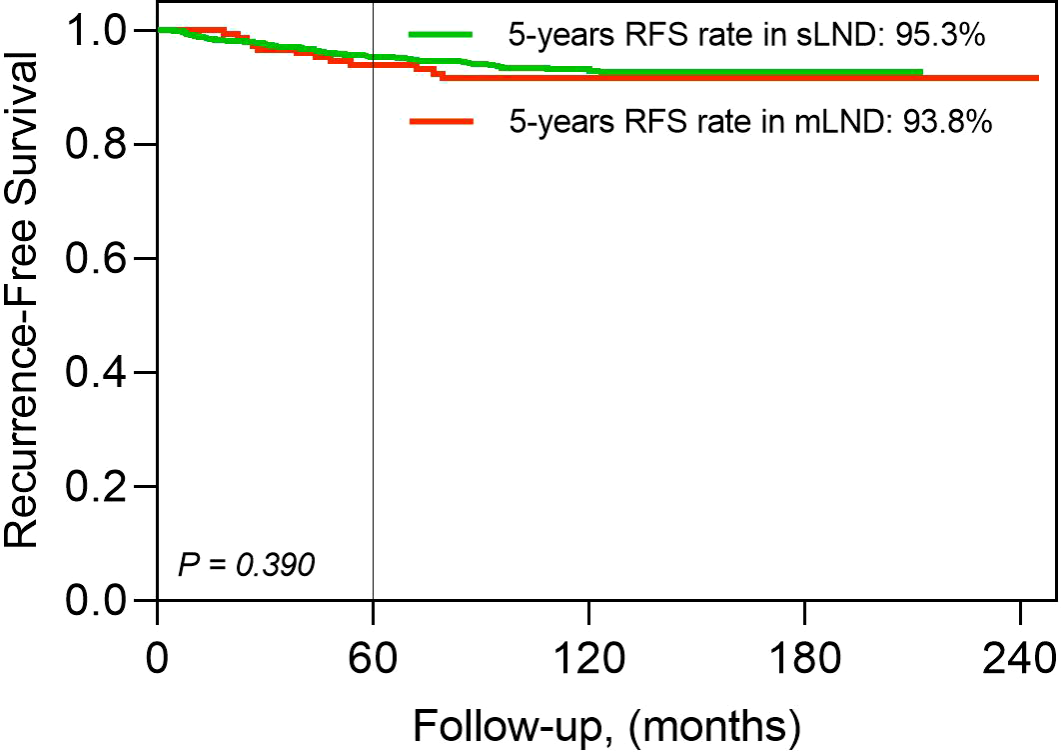
Kaplan–Meier analysis of recurrence-free survival (RFS) rates according to lateral neck dissection. mLND, metachronous lateral neck dissection; sLND, synchronous lateral neck dissection.

**Figure 3:**
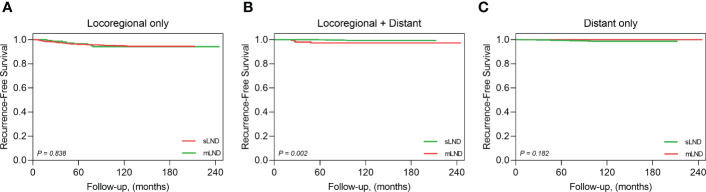
Kaplan-Meier analysis of recurrence-free survival by the recurrence site. **(A)** locoregional metastasis only, **(B)** locoregional and distant metastasis, **(C)** distant metastasis only.

Structural recurrence was confirmed in 110 patients, without any significant differences between the sLND and mLND groups (98 [6.1%] and 12 [8.2%], respectively, P=.32). The 5-year RFS rates in the sLND and mLND groups were 95.3% and 93.8%, respectively. **Figure 2** shows Kaplan–Meier analysis of RFS rates according to synchronous and metachronous LND. Of the 98 patients in the sLND group with structural recurrence, 74 had only locoregional recurrence, seven had locoregional recurrence with distant metastasis, and 17 had only distant metastasis. In the mLND group, among 12 cases of structural recurrence, eight had locoregional recurrence only, and four had locoregional recurrence with distant metastasis. **Figure 3** shows the Kaplan-Meier analysis of recurrence-free survival (RFS) in the sLND group and the mLND group according to the site of recurrence. Locoregional recurrence with distant metastasis showed a significant difference between the sLND and the mLND groups (P=.002). The period from surgery to recurrence was significantly longer in the mLND group than in the sLND group (115.4 [range 18.4–233.1] months and 86.4 [range 5.9–200.8] months, respectively, P<.001). The median follow-up period was significantly longer in the mLND group than in the sLND group (130.5 months [range 12.6–245.2] and 97.8 months [range 12.3–212.9 months], respectively, P<.001).

### 3.3 Risk factors for post-treatment recurrence in mLND group

**Figure 4:**
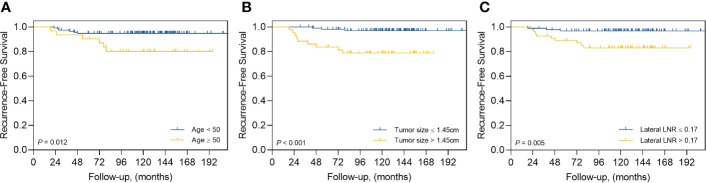
Kaplan–Meier curves estimating recurrence-free survival by **(A)** age at first thyroid surgery, **(B)** tumor size, and **(C)** lymph node ratio (LNR).

The results of the ROC curve and AUC analyses are presented in **Supplementary Figure 1**. The AUC and cutoff values for age at first thyroidectomy were 0.774 and 50 years, respectively (P=.002), and 0.781 and 1.45 cm for tumor size, respectively (P=.001). The AUC and cutoff values for the number of LNs were 0.659 and 40 for retrieved LNs in the lateral compartment, respectively (P=.05); 0.756 and 7 for metastatic LNs in the lateral compartment, respectively (P=.003); and 0.696 and 0.17 for the LN ratio in the lateral compartments, respectively (P=.03). The AUC and cutoff values for postoperative stimulated-Tg levels were 0.724 and 1.7 ng/mL, respectively, and 0.691 and 0.1 ng/mL for suppressed Tg levels (P=.01 and P=.03, respectively). For RFS, univariate analyses showed that age (P=.002), tumor size (>1.45 cm) (P=.002), the LN ratio in the lateral compartments (P=.012), and postoperative stimulated Tg levels (P=.034) were significantly associated with RFS (**Table 3**). In multivariate analyses, the age (adjusted HR=5.209, 95% CI=1.359–19.964; P=.02), tumor size (>1.45 cm) (adjusted HR=4.022, 95% CI=1.036–15.611; P=.04), and the LN ratio in the lateral compartment (adjusted HR=4.043, 95% CI=1.079–15.148; P=.04) were independent predictors of recurrence (**Table 4**). **Figure 4** shows the Kaplan–Meier curves estimating RFS according to the cutoff points of age, tumor size, and LN ratio in the lateral compartment.

**Table 3:** Univariate analyses of factors associated with RFS in the mLND group.

Variable	HR	HR 95% CI		p-value
		Lower limit	Upper limit	
Age, ≥50 years	7.806	2.113	28.836	.002
Sex, female	1.484	0.325	6.773	.61
Tumor size, >1.45 cm	8.013	2.169	29.607	.002
Tumor multifocality	1.194	0.385	3.703	.76
Extrathyroidal extension	1.403	0.422	4.659	.58
Chronic lymphocytic thyroiditis	0.680	0.149	3.105	.62
Central neck compartment				
Retrieved LN number, >10	1.255	0.378	4.169	.71
Metastatic LN number, >5	1.039	0.281	3.839	.95
Central LN ratio, >0.5	1.233	0.391	3.886	.72
Lateral neck compartment				
Retrieved LN number, >40	1.928	0.612	6.076	.26
Metastatic LN number, >7	1.164	0.350	3.866	.80
Lateral LN ratio, >0.17	5.288	1.431	19.536	.01
Stimulated Tg, >1.77 ng/dL	4.096	1.109	15.130	.03
Suppressed Tg, >0.1 ng/dL	2.855	0.906	8.995	.07

HR, hazard ratio; CI, Confidence interval; LN, lymph node; RFS, recurrence-free survival; mLND, metachronous lateral neck dissection.

**Table 4:** Multivariate analyses of factors associated with RFS in the mLND group.

Variable	HR	HR 95% CI		p-value
		Lower limit	Upper limit	
Age, ≥50 years	5.209	1.359	19.964	.02
Tumor size, >1.45 cm	4.022	1.036	15.611	.04
Lateral LN ratio, >0.17	4.043	1.079	15.148	.04

pTNM proposed by the American Joint Committee on Cancer (AJCC, 8th edition); LND, lateral neck dissection; LNs, lymph nodes; HR, hazard ratio; RFS, recurrence-free survival; HR, hazard ratio; CI, Confidence interval.

## 4 Discussion

Although cervical LN metastasis frequently occurs in patients with PTC, there is insufficient evidence that regional LN metastasis affects survival (12, 13). However, LN metastasis is associated with increased risk of local recurrence in patients with PTC (14, 15). Thyroid cancer recurrence after initial surgery has significant psychosocial implications, as patients require additional surgery and RAI treatment; reoperation along the previously incised plane is complex, with a high risk of morbidity and additional pressure on surgeons (11, 16).

This study focused on lateral lymph nodal recurrence in patients with PTC who previously underwent thyroidectomy without early clinical evidence of lateral LN metastasis. Previously, lateral neck metastases were found in 4.5% (11/246) of patients who underwent total thyroidectomy (17). Our results showed an overall recurrence rate of 6.3% during a median follow-up period of 102.1 months. During the study period at our institution, 17,779 patients underwent total and less than total thyroidectomy, of which 147 patients received mLND due to lateral lymph node recurrence. Later, lateral neck LN recurrence and distant metastases were found in only 8.2% (12/147) of the mLND group and 6.1% (87/1613) of the sLND group, showing a higher rate in the mLND group, without statistical significance. This suggests that preoperative imaging is adequate. Setting an appropriate limit to the extent of planned surgery to ensure that it is not excessive is important. The median period from LND to recurrence was significantly longer in the mLND group than in the sLND group, indicating a sufficiently long disease-free survival even with a minimal surgical range. Moreover, the average duration of 113.6 months in the mLND group suggests a long-term follow-up with precise radiological examination after LND.

In the mLND group, age at first thyroidectomy (≥50 years), primary tumor size (>1.45 cm), and LN ratio in the lateral compartment (>0.25) were independent factors for post-treatment recurrence. Thus, our results indicate that in the mLND group, age at time of surgery and examination of the primary tumor and cervical LNs are essential for identifying patients at risk for recurrence.

The number of metastatic LNs at diagnosis is associated with recurrence in PTC (15, 18). Additionally, the LN ratio (number of metastatic LNs divided by the number of harvested LNs) is associated with recurrence after treatment. In a study of 390 patients with PTC who underwent total thyroidectomy and lateral neck resection, recurrence was predicted by central neck LN ratio >0.44 and lateral neck LN ratio >0.29 (19). Another study reported that patients with an LN ratio >0.7 had significantly worse disease-free survival than those with an LN ratio <0.7 (20). However, another study found that central and lateral LN ratios were not associated with recurrence in patients who underwent central and lateral cervical resection (21). In the present study, there was no significant difference in lateral LN ratio between the mLND and sLND groups, and lateral LN ratio (>0.17) in the mLND group was an independent recurrence risk factor. Therefore, patients receiving mLND should be informed of the risk of recurrence, even if the rate of recurrence in lateral LN ratio is low. LN ratio is a valuable prognostic factor for disease-free survival; however, further multicenter studies are needed to establish optimal cutoffs and complement TNM staging.

In this study, age at diagnosis (≥50 years) was a risk factor for recurrence in the mLND group. The relationship between age at onset and prognosis of thyroid cancer has been extensively studied and applied to various cancer staging systems based on previous findings that the mortality associated with thyroid cancer increases rapidly from age 40 to 50 (22, 23). Forty-five years was used as a cutoff age for differentiating age-related survival in the AJCC Cancer Staging Manual, 7th edition in 2018, and the 8th edition raised the age cutoff from 45 to 55 years (24). The 50-year cutoff identified in this study is important for warning patients receiving mLND of potential recurrence.

We found tumor size (>1.45 cm) to be another risk factor for recurrence in the mLND group. The American Common Cancer Tumor Staging System for thyroid cancer uses size thresholds of 2 and 4 cm for T staging, with larger sizes associated with lower survival rates. Several studies have shown that a larger tumor size affects lateral LN metastasis (25, 26). The tumor size cutoff, which is considered a risk factor for lateral cervical metastasis, has reported values ranging from 0.5–1.5 cm (27–29), and a study showed that tumor size <2.5 cm did not affect mortality rate (30). Although the threshold of 1.45 cm used in the mLND group in this study differs from that of other studies, it suggests that follow-up for recurrence is necessary for mLND patients.

ETE and presence of CLT in the initial surgeries, which differed significantly between the sLND and mLND groups, confirmed that the sLND group had a more aggressive tendency at the time of diagnosis. ETE is an important prognostic factor for survival outcome in PTC. According to a recent AJCC recommendation, microscopic ETE was excluded from the definition of T3 disease (24). Therefore, tumors with an ETE affecting only the strap muscles are considered T3b (24). CLT is an autoimmune disease characterized by fibrosis, atrophy, and lymphocyte infiltration of thyroid tissue. It is caused by a thyroid-specific antigen in thyroid tumors that may be involved in destroying thyroid cancer. Several studies have shown that patients with PTC coexisting with CLT have a lower recurrence rate and better overall survival due to better-controlled tumor growth and proliferation (31, 32). However, in this study, LND recurrence between the two groups did not differ significantly, suggesting that appropriate additional treatment is only required in cases of recurrence.

This study has several limitations. The study design was retrospective and conducted at a single institution. In addition, we included data from patients who underwent operations performed by several surgeons in our institution. Moreover, the different aggressive characteristics of the sLND and mLND groups at the time of PTC diagnosis may have influenced their outcomes. ETE in this study includes both microscopic and gross ETE. Multifocality was also defined as the presence of two or more isolated/non-contiguous tumor foci of any size in the resected thyroid gland. According to the recent AJCC recommendation, microscopic ETE was excluded from the definition of T3 disease but included as necessary for evaluation in mLND patients and will be studied separately in future studies. Nevertheless, this study is meaningful in that the effects of minimizing the extent of surgery with a thorough preoperative examination were evaluated.

The results of the present study showed no difference in recurrence between the mLND and sLND groups. Therefore, mLND is a suitable treatment option for PTC in terms of recurrence, and additional treatment is only required in cases of recurrence after LND. The age (≥50 years), tumor size (>1.45 cm), and lateral LN ratio (>0.17) were strong predictors of RFS in the mLND group, suggesting the importance of careful post-treatment monitoring of patients with these risk factors.

## Data availability statement

The raw data supporting the conclusions of this article will be made available by the authors, without undue reservation.

## Ethics statement

The studies involving human participants were reviewed and approved by The Institutional Review Board of Gangnam Severance Hospital, Yonsei University College of Medicine (Seoul, Korea). Written informed consent for participation was not required for this study in accordance with the national legislation and the institutional requirements.

## Author contributions

Concept and design: YL and HY; Acquisition, analysis, or interpretation of data: HY, JiSL, and HSC; Drafting of the manuscript: HY, YL, and S-MK; Critical revision of the manuscript for important intellectual content: All authors; Statistical analysis: HY and JuSL; Administrative, technical, or material support: YL, S-MK, HSC, and CP; Supervision: YL, S-MK, HJC, HSC, and CP. All authors contributed to the article and approved the submitted version.
