# Supplementary material for: Lateral neck dissection for the treatment of synchronous and metachronous lateral neck metastasis of N1b papillary thyroid cancer
Source: Front Endocrinol (Lausanne). 2023 Jun 22;14:1166640. doi: 10.3389/fendo.2023.1166640 (PMC10325561; doi:10.3389/fendo.2023.1166640)
Supplement: Supplementary file 1 [file Image_1.pdf]

Supplementary Material

Lateral neck dissection for the treatment of synchronous and metachronous lateral neck metastasis of N1b papillary thyroid cancer

Hyeok Jun Yun, Jin Seok Lee, Jun Sung Lee, Seok Mo Kim, Hojin Chang, Yong Sang Lee*, Hang-Seok Chang, Cheong Soo Park

*** Correspondence:** Yong Sang Lee, MD, PhD, medilys@yuhs.ac

1 Supplementary Figure

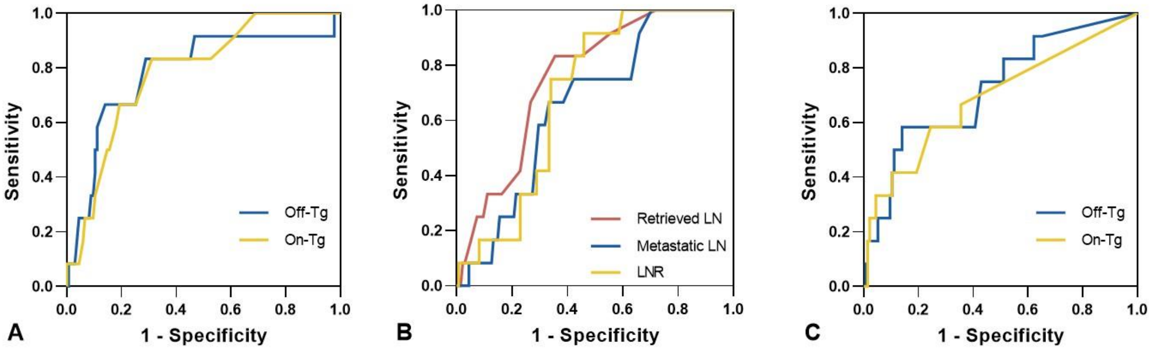

	AUC	SE	P	95% CI
Age	0.774	0.079	0.002	0.619-0.929
Tumor size	0.781	0.061	0.001	0.661-0.900

LND	AUC	SE	P	95% CI
Retrieved	0.659	0.066	0.048	0.530-0.788
Metastatic	0.756	0.058	0.003	0.643-0.869
Ratio	0.696	0.054	0.025	0.590-0.801

Tg	AUC	SE	P	95% CI
Off-Tg	0.724	0.079	0.010	0.570-0.879
On-Tg	0.691	0.089	0.028	0.518-0.865

Supplementary Figure 1. ROC curve analyses of age, tumor size, lateral lymph node, and thyroglobulin (Tg) levels predictive of RFS after mLND. The ROC AUCs were determined to estimate RFS according to age and tumor size **(A)**, lateral lymph node **(B)**, and Tg levels **(C)**. The off-Tg and on-Tg levels represent conditions with and without the stimulation of thyroid-stimulating hormone, respectively, at the time of radioactive iodine ablation (RAI) therapy just after thyroidectomy.

Abbreviations: AUC: area under the curve; CI: confidence interval; LN, lymph node; LND, lateral neck dissection; LNR, lymph node ratio; mLND: metachronous lateral neck dissection; RFS: recurrence-free survival; ROC: receiver operating characteristic; SE: standard error; .
